# Culture Negative Stent Infection in an Infant with Hypoplastic Left Heart and Persistent Fever

**DOI:** 10.1155/2015/496108

**Published:** 2015-09-08

**Authors:** Kurt D. Piggott, Federico Laham, Alejandro Jordan Villegas, Harun Fakioglu, Carlos Blanco, Sukumar Suguna Narasimhulu

**Affiliations:** ^1^The Heart Center at Arnold Palmer Hospital for Children, Pediatric Cardiac Intensive Care, 92 W. Miller Street, MP 307, Orlando, FL 32806, USA; ^2^Arnold Palmer Hospital for Children, Pediatric Infectious Disease, USA

## Abstract

We present an infant with hypoplastic left heart with persistent fever despite two courses of antibiotics and repeatedly negative blood cultures. He eventually underwent surgical extraction of two stents. The stent cultures became positive; he was treated with 4 weeks of antibiotics and the fever resolved.

## 1. Background

Fever is commonly seen in patients following surgery for congenital heart disease. The frequency of fever following cardiovascular surgery has been reported between 12% and 73% [1]. A source may not be identified, and if persistent the fever can become especially troublesome [2]. This may be further complicated when the patient has an artificial valve or endovascular stents. Duration of antibiotic therapy is often determined by culture results and clinical status of the patient. We report a case of an infant with hypoplastic left heart and persistent fever who despite numerous negative blood cultures had evidence of stent-associated infection which was confirmed by direct culture after removal. We also discuss the management.

## 2. Case Report

The case is a 4-month-old male born with mitral and aortic stenosis variant of hypoplastic left heart syndrome. He underwent a Norwood procedure with placement of a 4.0 mm modified Blalock-Taussig shunt. He had a difficult postoperative course plagued by low cardiac output syndrome, vocal cord paralysis, and several failed extubations. One month postoperatively he required cardiac catheterization and placement of a stent in the distal systemic-pulmonary artery shunt due to stenosis and acute desaturation. Two months postoperatively he required stent placement in the interatrial septum due to a progressively increasing gradient by echocardiogram. One month later he was discharged home. He returned 36 hours later with a fever of 106 Fahrenheit (F) and clinical shock. His white blood cell (WBC) count was 21,000 cells/mL with a procalcitonin of 212 ng/mL and c-reactive protein (CRP) of <0.5 mg/dL. He was pan-cultured and placed on broad-spectrum antibiotics. All cultures remained negative, including viral studies. He was treated for 14 days with vancomycin and cefepime for presumed culture negative septic shock. He was discharged home 48 hours after completion of antibiotics. He returned 24 hours later with fever of 101.3 Fahrenheit (F). Several blood culture sets were obtained and he was observed off antibiotics for 48 hours during which time he remained afebrile, so he was discharged home. He again returned 24 hours later with fever of 101.9 F. Blood and urine cultures were again performed, his WBC count was 23,600 cells/mL, and CRP was 3.6 mg/dL. He was observed off antibiotics for 24 hours but the fever curve was increasing so a central venous line (CVL) was placed, new cultures from the line were drawn, and the patient was placed on broad-spectrum antibiotics. The line cultures grew the following day and were identified as* Staphylococcus epidermidis*, with a vancomycin minimal inhibitory concentration (MIC) of 4 mcg/mL (Table 1). Cultures from both CVL lumens were repeated the following day and both grew the same organisms that evening. Vancomycin was continued for 10 days. Repeat cultures remained negative and he remained afebrile and the CVL was removed. Two days after vancomycin was stopped he began to spike fevers again. Daily blood cultures were drawn and were repeatedly sterile. He had normal CBC and CRP values. He remained clinically well and was kept off antibiotics. Possible etiologies were discussed and included infected stents, central fever, and fever related to low cardiac output. We discussed the possibility of culture negative endocarditis; however, he did not meet the major or minor Duke criteria for diagnosis. After much discussion, he was placed on vancomycin 24 hours prior to surgery. He underwent a Glenn procedure and removal of all stents, which were subsequently sent for culture. Cultures from both stents grew bacteria less than 12 hours after removal. The stent from the shunt grew* Staphylococcus warneri* and* Staphylococcus epidermidis*. Vancomycin MIC were 1 and 2 mcg/mL, respectively (Table 2). The stent from the atrial septum grew* Staphylococcus epidermidis*, with a different antibiotic susceptibility profile than the one isolated previously from the CVL and therefore was felt to be a different strain (Table 3). The patient was treated for 6 weeks with intravenous vancomycin and oral rifampin, in combination with gentamicin for the first two weeks. The blood cultures remained negative, and he remained afebrile and clinically well. He was discharged 5 days after antibiotics were completed. He remains symptom-free as documented in a follow-up visit 1 month after discharge.

## 3. Discussion

Fever is a common occurrence in pediatric patients but becomes increasingly cumbersome when the patient is critically ill, after cardiac surgery, or after cardiac catheterization with stent implantation. With the onset of fever, WBC count, CRP or procalcitonin, and blood cultures are typically obtained and empiric antibiotics are commenced. However, in young infants and the early postoperative patient, these inflammatory markers may be unreliable [3]. If cultures remain negative and fever continues without an obvious source of infection, determining the duration of antibiotics becomes more difficult. In cases where there is clinical deterioration or concern, it may be feasible to treat for 7–10 days, typically with the input of clinical markers and infectious disease guidance.

The above patient is unique due to the persistence of fever and clinical deterioration despite negative blood cultures. He received two separate treatment courses with vancomycin. On both occasions the fever resolved, only to return following antibiotic cessation. He continued with fever for 10 days off antibiotics with daily negative cultures, normal inflammatory markers, and unremarkable physical exam. After much discussion we suspected that the stents were likely colonized/infected and that they should be removed and would do so at the time of the Bidirectional Glenn operation. We also decided that we would monitor the patient closely “off antibiotics” in the hospital with the goal of increasing the culture yield. Per surgical request, vancomycin was commenced 24 hours prior to surgery. As stated above, the cultures from both stents became positive in less than 12 hours. Interestingly, the susceptibility profiles of the bacteria that grew from the stents were different from those that grew from the CVL. Unfortunately, the original* Staphylococcus epidermidis* isolate was unavailable to perform pulse-field gel electrophoresis, to determine if it was an identical strain. The fevers disappeared following surgery and a prolonged antibiotic treatment regimen, similar to the one provided for endocarditis with the presence of prosthetic material.

We believe that this complex infectious disease case is important to report. A recent case series of 85 adults with vascular grafts (the majority for lower limb arterioplasty) found that preoperative blood cultures were positive in only 29 of the 78 patients with an identified organism [4]. In retrospect, the indolent course is consistent with an infection of an endovascular device with a low virulence organism such as coagulase-negative staphylococci. However, his presentation in shock clouded this picture. Our current hypothesis is that the stents were likely seeded following removal of his initial CVL and the infection was festering and did not manifest clinically until after discharge. The present case underscores the need to exclude a stent-associated infection in the setting of persistent fever, normal inflammatory markers, and negative blood cultures and may require surgical extraction for treatment.

## Figures and Tables

**Table 1:** Blood culture results and susceptibility profile for *Staphylococcus epidermidis* isolated from the central venous catheter.

Antibiotic	MIC	Susceptibility
Daptomycin	0.5	Susceptible
Doxycycline	1	Susceptible
Erythromycin	≤0.25	Susceptible
Oxacillin	≥4	Resistant
Vancomycin	4	Susceptible
Trimeth-Sulfa	≤10	Susceptible

MIC = minimum inhibitory concentration, Trimeth-Sulfa = trimethoprim-sulfamethoxazole.

**Table 2:** Blood culture results and susceptibility profile for the *Staphylococcus epidermidis* and *Staphylococcus warneri* isolated from the stent in the systemic-pulmonary artery shunt.

Organism	Antibiotic	MIC	Susceptibility
*Staph epidermidis*	Daptomycin	0.5	Susceptible
	Doxycycline	≥16	Resistant
	Erythromycin	≤0.25	Susceptible
	Oxacillin	≥4	Resistant
	Vancomycin	2	Susceptible
	Gentamicin	≤0.5	Susceptible
	Rifampin	≤0.5	Susceptible

*Staph warneri*	Daptomycin	1	Susceptible
	Doxycycline	≤0.5	Susceptible
	Erythromycin	≥8	Resistant
	Oxacillin	≤0.25	Susceptible
	Vancomycin	1	Susceptible
	Gentamicin	≤0.5	Susceptible
	Rifampin	≤0.5	Susceptible

MIC = minimum inhibitory concentration.

**Table 3:** Blood culture results and susceptibility profile for *Staphylococcus epidermidis* isolated from the atrial septal stent.

Antibiotic	MIC	Susceptibility
Daptomycin	1	Susceptible
Doxycycline	≤0.5	Susceptible
Erythromycin	≥8	Resistant
Oxacillin	≤0.25	Susceptible
Vancomycin	1	Susceptible

MIC = minimum inhibitory concentration.
